# Kinder mit akuter Atemwegsinfektion in Kinderkliniken im Herbst/Winter 2020/21: Empfehlungen der Deutschen Gesellschaft für Pädiatrische Infektiologie (DGPI) und der Deutschen Gesellschaft für Krankenhaushygiene (DGKH) (Stand 14.10.2020)

**DOI:** 10.1007/s00112-020-01058-9

**Published:** 2020-11-06

**Authors:** Reinhard Berner, Johannes Huebner, Hans-Iko Huppertz, Arne Simon, Peter Walger, Deutsche Gesellschaft für Pädiatrische Infektiologie (DGPI), Deutsche Gesellschaft für Krankenhaushygiene e. V. (DGKH)

**Affiliations:** 1 Klinik und Poliklinik für Kinder- und Jugendmedizin, Universitätsklinikum Carl Gustav Carus an der Technischen Universität Dresden, Fetscherstr. 74, 01307 Dresden, Deutschland; 2 Abteilung Pädiatrische Infektiologie, Dr. von Haunersches Kinderspital, Klinikum der Ludwig-Maximilian-Universität München, München, Deutschland; 3 Bremen, Deutschland; 4 Klinik für Pädiatrische Onkologie und Hämatologie, Universitätsklinikum des Saarlandes, Homburg/Saar, Deutschland; 5 Deutsche Gesellschaft für Krankenhaushygiene e. V. (DGKH), Berlin, Deutschland

**Keywords:** SARS-CoV‑2, Atemwegsinfektion, Hospitalisierung, Kohortierung, Isolierung, Kinder und Jugendliche, SARS-CoV‑2, Respiratory tract infection, Hospitalization, Isolation, Cohorting, Children and adolescents

## Abstract

Im Herbst und Winter 2020/2021 wird der Umgang mit Fällen einer vermuteten oder nachgewiesenen SARS-CoV-2-Infektion die bereits durch die bekannten saisonalen Virusinfektionen (u. a. RSV [respiratory syncytial virus] oder Influenzavirus) oft über die Grenzen hinaus belasteten Kinder- und Jugendkliniken hinsichtlich Isolierung oder Kohortierung vor erhebliche zusätzliche Herausforderungen stellen. Dies wird zu Engpässen in der Versorgung nicht nur der akut kranken Kinder, sondern mutmaßlich auch vieler anderer Patientenpopulationen führen. Daher müssen pragmatische, aber gleichwohl krankenhaushygienisch vertretbare Lösungsansätze vorbereitet werden. Die hier erarbeitete Stellungnahme soll den Kinderkliniken Hilfestellung für die Entscheidungsfindung geben. Diese orientiert sich im Wesentlichen an der Inzidenz von SARS-CoV‑2 im Kreis bzw. im Einzugsgebiet. Ist die Inzidenz niedrig (kumulative Anzahl der Neuinfektionen in den letzten 7 Tagen: <25/100.000) oder mittelhoch (25–50/100.000), erfolgt die Diagnostik unter Standardhygienebedingungen und die Kohortierung je nach Erreger oder Symptomatik. Ist die Inzidenz der Neuerkrankungen in der Region hoch (>50/100.000), erfolgt die Diagnostik unter entsprechenden, vom RKI vorgegebenen Sicherheitsvorkehrungen mit nachfolgender Isolierung bzw. Kohortierung der Patienten. Die vorgeschlagenen Grenzwerte können nach Maßgabe der Gesundheitsbehörde variieren. Bei überschrittener Aufnahmekapazität oder Versorgungsengpässen ist im Einzelfall die akute Notwendigkeit einer medizinischen Notfallversorgung prioritär, was vorübergehend ein weniger striktes Isolierungsregime erfordern kann.

## Abstract (en)

Due to the shortage of pediatric hospital beds in general and due to the large annual burden of hospital admissions for common respiratory tract infections, such as influenza or RSV [respiratory syncytial virus] in particular, it can be expected that hospitalization of children with suspected or confirmed SARS-CoV‑2 infections will face hospitals with an even more tense situation than usual in the winter 2020/2021. This tenuous shortage may touch various aspects but in the first place it will affect isolation and cohorting. In addition, this shortage will not only apply to acutely ill children with viral respiratory tract infections but all children with need for hospital care, either chronically ill or e.g. being premature babies or newborns. Therefore, approaches are required which on the one hand are based on pragmatic grounds but on the other hand fulfill the basics of hygiene and infection control. The recommendations proposed in this statement are intended to give assistance to hospitals for the management of testing, isolation and cohorting of pediatric patients with suspected or confirmed SARS-CoV‑2 infections. The most important factor navigating the essential measures is the cumulative incidence of newly diagnosed infections per 100,000 over the last 7 days, which is given by the RKI or the local public health authorities. In the situation of low (<25/100,000) or medium (25–50/100,000) incidence the respective diagnostic measures and hospital admission can be performed under standard hygiene precautions and the children will be cohorted according to their clinical presentation until the results of SARS-CoV‑2 test (or RSV [respiratory syncytial virus] or influenza test) are available. In the situation of high (>50/100,000) incidence the respective diagnostic measures and the admission have to be performed under SARS-CoV‑2 precautions as specified by the RKI, and the children have to be isolated until the results of SARS-CoV‑2 test are available. The assessment of the incidence figures and the respective measures may be adapted by the local public health authorities on an individual basis. In case of shortfalls in admission capacities, the requirement of acute emergency care may necessitate that isolation and cohorting in the hospital will have to be performed temporarily in a less restrictive way than recommended here for standard.

Im Herbst und Winter 2020/2021 wird mit großer Wahrscheinlichkeit die Bettenkapazität in den Kinder- und Jugendkliniken noch knapper werden als in den Vorjahren. In den Herbst- und Wintermonaten wird – in Abhängigkeit vom Lebensalter – eine voraussehbar große Zahl von Kindern und Jugendlichen in Kinderkliniken wegen Atemwegsinfektionen hospitalisiert werden. Der Umgang mit Fällen einer vermuteten oder nachgewiesenen SARS-CoV-2-Infektion in Bezug auf Isolierung oder ggf. Kohortierung wird die bereits durch die üblichen saisonalen Virusinfektionen (u. a. RSV [respiratory syncytial virus] oder Influenzavirus) oft über die Grenzen hinaus belasteten Kinder- und Jugendkliniken vor erhebliche zusätzliche Herausforderungen stellen.

Dies wird zu Engpässen in der Versorgung nicht nur der akut erkrankten Kinder, sondern mutmaßlich auch von chronisch kranken und chirurgischen Patienten wie auch von Neu- und Frühgeborenen führen. Eine Erweiterung von Kapazitäten durch Bereitstellung zusätzlicher Betten wird im Allgemeinen – nicht zuletzt aufgrund von Personalmangel bei Ärzten und Pflegepersonal – nicht realisierbar sein. Regional müssen frühzeitig Vorbereitungen und Absprachen zwischen benachbarten Kliniken und mit den niedergelassenen Kinder- und Jugendärztinnen und -ärzten getroffen werden. Gegebenenfalls sind in diese Vereinbarungen auch die Krankenhausdirektionen, die Kassenärztliche Vereinigung und die Gesundheitsämter einzubeziehen. Grundsätzlich müssen pragmatische, gleichwohl aber krankenhaushygienisch vertretbare Lösungsansätze für den Umgang mit vermuteten oder nachgewiesenen SARS-CoV-2-Infektionsfällen vorbereitet werden.

Die Überlegungen zur Kapazitätsüberschreitung der Kinder- und Jugendkliniken sind auch vor dem Hintergrund des seit Jahren bestehenden Personalmangels zu betrachten. Beschäftigte im Gesundheitswesen und insbesondere in Kinder- und Jugendkliniken sind einem erhöhten Risiko einer Infektion mit SARS-CoV‑2 während ihrer beruflichen Tätigkeit ausgesetzt. Der effektive Schutz der am Patienten tätigen Mitarbeiter ist daher eine der wichtigsten Voraussetzungen zur Aufrechterhaltung des Betriebs einer Kinderabteilung unter den zu erwartenden Herausforderungen der kommenden Herbst‑/Wintersaison.

Aktuell werden Kinder und Jugendliche mit respiratorischen, gastrointestinalen oder weiteren Symptomen, die auf eine schwere systemische Infektion hinweisen, sowie Patienten mit Geruchs- oder Geschmacksstörungen mittels Rachen- oder Nasopharyngealabstrich regelhaft auf eine SARS-CoV-2-Infektion getestet. In der Vergangenheit waren hierfür ausschließlich PCR-Verfahren verfügbar. Hier variiert die Zeit zwischen Abstrichentnahme und Ergebnismitteilung in der Realität zwischen wenigen Stunden und mehreren Tagen je nach örtlichen Gegebenheiten. Bis zum Erhalt des Testergebnisses bleibt der Verdacht auf eine SARS-CoV-2-Infektion bestehen, und der hospitalisierte Patient muss entsprechend isoliert oder kohortiert werden. In naher Zukunft werden Antigenschnelltests mit akzeptabler Testperformance (Sensitivität und Spezifität) oder auch PCR-Tests als Point-of-care-Diagnostik ein Analyseergebnis innerhalb 1 h verfügbar machen. Die Anwendung von Schnelltests könnte den Umgang mit dieser Situation speziell in der Kinder- und Jugendmedizin erheblich erleichtern. Bezüglich Verfügbarkeit und den Modalitäten der Anwendung sowie der Testperformance in der pädiatrischen Population bestehen allerdings noch beträchtliche Unsicherheiten.

Wegen der Kozirkulation mit anderen respiratorischen Erregern, die als Verursacher der akuten Infektionssymptomatik bei den betroffenen Kindern bei Weitem häufiger auftreten werden als SARS-CoV‑2, sollten alle Kinder mit entsprechenden Symptomen bei Aufnahme ins Krankenhaus in Abhängigkeit von der regionalen und jahreszeitlichen Inzidenz auf spezifische respiratorische Erreger (RSV, Influenza) getestet werden, um so die Kohortierung der Patienten steuern zu können. Wenn das Kind typische Krankheitssymptome von COVID-19 (z. B. Geruchs- oder Geschmacksstörungen) aufweist oder eine entsprechende Anamnese eines direkten Kontakts mit einem Patienten mit nachgewiesener SARS-CoV-2-Infektion vorliegt, erfolgen die Abstrichentnahme unter entsprechenden Sicherheitsvorkehrungen (FFP2-Maske, Schutzkittel, Handschuhe, Brille) sowie die Isolierung des Patienten bis zum Erhalt des Testergebnisses; das weitere Vorgehen wird je nach Testergebnis festgelegt: Positiv getestete Kinder werden isoliert oder kohortiert unter den entsprechenden etablierten Hygienestandards (https://www.rki.de/DE/Content/InfAZ/N/Neuartiges_Coronavirus/Hygiene.html).

In allen übrigen Situationen orientiert sich die Versorgung der Kinder an der Inzidenz von SARS-CoV‑2 im Kreis bzw. im Einzugsgebiet. Ist die Inzidenz niedrig (kumulative Anzahl der Neuinfektionen in den letzten 7 Tagen: <25/100.000) oder mittelhoch (25–50/100.000), erfolgt die Abstrichentnahme unter *Standardhygienebedingungen* (Mund-Nasen-Schutz [Medizinprodukt] und Schutzbrille oder Visier). Die Kohortierung erfolgt je nach Erreger oder Symptomatik. Wenn die Inzidenz der Neuerkrankungen in der Region hoch ist (>50/100.000), erfolgen die Abstrichentnahme unter entsprechenden *Sicherheitsvorkehrungen* sowie die Isolierung des Patienten bzw. Kohortierung.

Bei der Aufnahme auf Stationen mit besonders vulnerablen Patienten wie Kinderonkologie, Intensivstation oder Kinderkardiologie kann es ggf. sinnvoll sein, auch bei niedrigerer Inzidenz im Kreis alle, d. h. auch elektiv aufzunehmende Kinder abzustreichen und zunächst zu isolieren, bis das Abstrichergebnis vorliegt. Dies ist nach ärztlicher Risikoanalyse vor Ort festzulegen.

Die Tab. 1 beschreibt die wichtigsten Szenarien und konkretisiert das Vorgehen im Hinblick auf die Abstrichentnahme und die Hygienekonzepte wie Isolierung oder Kohortierung.

**Table 1:** 

Status vor Aufnahme	7‑Tage-Inzidenz im Kreis^a^	Abstrichentnahme	Aufnahmebedingungen^b^
Auf COVID-19 hinweisende Symptome oder Kontakt mit nachgewiesener SARS-CoV-2-Infektion	Kein Einfluss	Unter Sicherheitsvorkehrungen	Isolierung, bis Abstrichergebnis vorliegt
(Unspezifische) Symptome einer akuten Infektionskrankheit^c^	≤50/100.000	Unter Standardbedingungen	Aufnahme mit syndromaler Kohortierung
	>50/100.000	Unter Sicherheitsvorkehrungen	Isolierung, bis Abstrichergebnis vorliegt
Bereits hospitalisierte Patienten mit neuen Symptomen einer Atemwegsinfektion	≤50/100.000	Unter Standardbedingungen	Isolierung oder syndromale Kohortierung, bis Abstrichergebnis vorliegt
	>50/100.000	Unter Sicherheitsvorkehrungen	Isolierung oder Kohortierung, bis Abstrichergebnis vorliegt

^a^Die in dieser Spalte vorgeschlagenen Grenzwerte können nach Maßgabe der regionalen Gesundheitsbehörde variieren

^b^Bei überschrittener Aufnahmekapazität oder Versorgungsengpässen ist im Einzelfall die akute Notwendigkeit einer medizinischen Notfallversorgung prioritär, was vorübergehend ein weniger striktes Isolierungsregime erfordern kann

^c^Fieber und/oder Symptome vereinbar mit einer akuten Atemwegsinfektion oder gastrointestinale Symptome, sofern hier nicht die eindeutige Diagnose einer bakteriellen Infektion (z. B. Tonsillopharyngitis durch A‑Streptokokken, Harnwegsinfektion) gestellt werden kann

^d^Mit aufgenommene Begleitpersonen von Kindern müssen anhand der regionalen epidemiologischen Situation befragt und ggf. getestet werden – wie für elektive Patienten im jeweiligen Krankenhaus und vom zuständigen Gesundheitsamt vorgesehen (s. oben). Für Begleitpersonen gelten die gleichen Hygiene- und Isolierungsmaßnahmen wie für ihre Kinder

